# Stereotactically Injected Kv1.2 and CASPR2 Antisera Cause Differential Effects on CA1 Synaptic and Cellular Excitability, but Both Enhance the Vulnerability to Pro-epileptic Conditions

**DOI:** 10.3389/fnsyn.2020.00013

**Published:** 2020-03-25

**Authors:** Timo Kirschstein, Erika Sadkiewicz, Gerda Hund-Göschel, Juliane Becker, Xiati Guli, Steffen Müller, Marco Rohde, Dora-Charlotte Hübner, Hannes Brehme, Stephan Kolbaske, Katrin Porath, Tina Sellmann, Annette Großmann, Matthias Wittstock, Steffen Syrbe, Alexander Storch, Rüdiger Köhling

**Affiliations:** ^1^Oscar Langendorff Institute of Physiology, University of Rostock, Rostock, Germany; ^2^Department of Neurology, University of Rostock, Rostock, Germany; ^3^Center of Transdisciplinary Neurosciences Rostock, University of Rostock, Rostock, Germany; ^4^Institute of Diagnostic and Intervention Radiology, University of Rostock, Rostock, Germany; ^5^Clinik for Pediatric and Adolescent Medicine, University of Heidelberg, Heidelberg, Germany

**Keywords:** limbic encephalitis, voltage-gated potassium channel, synaptic transmission, long-term potentiation, spike frequency adaptation

## Abstract

**Purpose:**

We present a case of voltage-gated potassium channel (VGKC) complex antibody-positive limbic encephalitis (LE) harboring autoantibodies against Kv1.2. Since the patient responded well to immunotherapy, the autoantibodies were regarded as pathogenic. We aimed to characterize the pathophysiological role of this antibody in comparison to an antibody against the VGKC-associated protein contactin-associated protein-2 (CASPR2).

**Methods:**

Stereotactic injection of patient sera (anti-Kv1.2-associated LE or anti-CASPR2 encephalopathy) and a control subject was performed into the hippocampus of the anesthetized rat *in vivo*, and hippocampal slices were prepared for electrophysiological purposes. Using extra- and intracellular techniques, synaptic transmission, long-term potentiation (LTP) and vulnerability to pro-epileptic conditions were analyzed.

**Results:**

We observed that the slope of the field excitatory postsynaptic potential (fEPSP) was significantly increased at Schaffer collateral-CA1 synapses in anti-Kv1.2-treated and anti-CASPR2-treated rats, but not at medial perforant path-dentate gyrus synapses. The increase of the fEPSP slope in CA1 was accompanied by a decrease of the paired-pulse ratio in anti-Kv1.2, but not in anti-CASPR2 tissue, indicating presynaptic site of anti-Kv1.2. In addition, anti-Kv1.2 tissue showed enhanced LTP in CA1, but dentate gyrus LTP remained unaltered. Importantly, LTP in slices from anti-CASPR2-treated animals did not differ from control values. Intracellular recordings from CA1 neurons revealed that the resting membrane potential and a single action potential were not different between anti-Kv1.2 and control tissue. However, when the depolarization was prolonged, the number of action potentials elicited was reduced in anti-Kv1.2-treated tissue compared to both control and anti-CASPR2 tissue. In contrast, polyspike discharges induced by removal of Mg^2+^ occurred earlier and more frequently in both patient sera compared to control.

**Conclusion:**

Patient serum containing anti-Kv1.2 facilitates presynaptic transmitter release as well as postsynaptic depolarization at the Schaffer-collateral-CA1 synapse, but not in the dentate gyrus. As a consequence, both synaptic transmission and LTP in CA1 are facilitated and action potential firing is altered. In contrast, anti-CASPR2 leads to increased postsynaptic potentials, but without changing LTP or firing properties suggesting that anti-Kv1.2 and anti-CASPR2 differ in their cellular effects. Both patient sera alter susceptibility to epileptic conditions, but presumably by different mechanisms.

## Introduction

Twenty years ago, limbic encephalitis (LE) associated with antibodies against voltage-gated potassium channels (VGKC) was described as a potentially reversible autoimmune encephalitis responding to immunotherapies which was in striking contrast to the formerly known forms of paraneoplastic encephalitis associated with antibodies against intracellular antigens (Buckley et al., 2001). Since VGKC antibodies interfered with the binding of the Kv1 channel blocker dendrotoxin (Hart et al., 2002), VGKC antibodies were believed to bind Kv1 channels. However, it turned out that VGKC antibodies were raised against Kv1-channel complex proteins such as leucine-rich, glioma inactivated 1 (LGI1) and contactin-associated protein-2 (CASPR2) (Lai et al., 2010; Irani et al., 2010). Recently, a large series of sera with VGKC complex antibodies were re-analyzed and only 56% harbored antibodies against LGI1 or CASPR2 (Lang et al., 2017). Almost all of the remaining sera, called double-negative VGKC complex antibody-positive samples, had cytosolic targets, but one serum bound to live hippocampal neurons, suggesting a possible novel surface antigen (Lang et al., 2017).

In this study, we present a case with LE admitted to our emergency presenting with status epilepticus. The patient’s serum was double-negative VGKC complex antibody-positive. Further analysis by a commercial laboratory revealed anti-Kv1.2, i.e., a pore-forming subunit of the VGKC Kv1. Since the patient responded well to immunotherapy, the autoantibodies were regarded as pathogenic in nature. In the hippocampus, Kv1.2 channels are particularly present in the middle molecular layer of the dentate gyrus, the CA1 stratum radiatum, and the CA1 stratum lacunosum-moleculare with the most striking immunoreactivity observed on axons and presynaptic terminals (Wang et al., 1993, 1994; Sheng et al., 1994; Monaghan et al., 2001; Gu et al., 2003; Wenzel et al., 2007; Lorincz and Nusser, 2008). Dendritic, but not somatic expression was also reported for cortical and hippocampal pyramidal cells (Sheng et al., 1994; Guan et al., 2006). In contrast, dentate granule cells appeared to be immunonegative (Sheng et al., 1994), and mossy fibers as well as CA3 pyramidal neurons seem to express Kv1.2 only during ontogenesis at immature stages (Prüss et al., 2010).

Functionally, Kv1.2 encodes a slowly activating and slowly inactivating D-type K^+^ conductance when expressed in Xenopus oocytes (Stühmer et al., 1989). D-type K^+^ currents in central neurons are primarily mediated by Kv1.1 and Kv1.2, both members of the Kv1 channel subfamily. They were recorded from CA1 neurons, and the activation and inactivation curves of D-type K^+^ currents cross near the resting membrane potential (RMP) giving rise to a window current (Storm, 1988), hence making this current powerfully regulate the neuron’s action potential threshold (Golding et al., 1999; Cudmore et al., 2010). The slow inactivation properties of Kv1 channels, in turn, contribute to spike repolarization (Mitterdorfer and Bean, 2002). Although Kv1.1 is much more widely expressed in the hippocampus than Kv1.2 (Wang et al., 1993, 1994; Veh et al., 1995; Grigg et al., 2000), Kv1.2 knock-out mice die after 15–18 days and are more prone to seizures than mice lacking Kv1.1 (Smart et al., 1998; Brew et al., 2007) supporting the view that Kv1.2 channels are crucial for hippocampal functional integrity. In this study, we injected patient sera containing anti-Kv1.2 or anti-CASPR2 into the rat hippocampus and asked whether synaptic or intrinsic properties were altered in the tissue from these rats.

## Materials and Methods

### Case of Kv1.2-Associated Limbic Encephalitis

**FIGURE 1:**
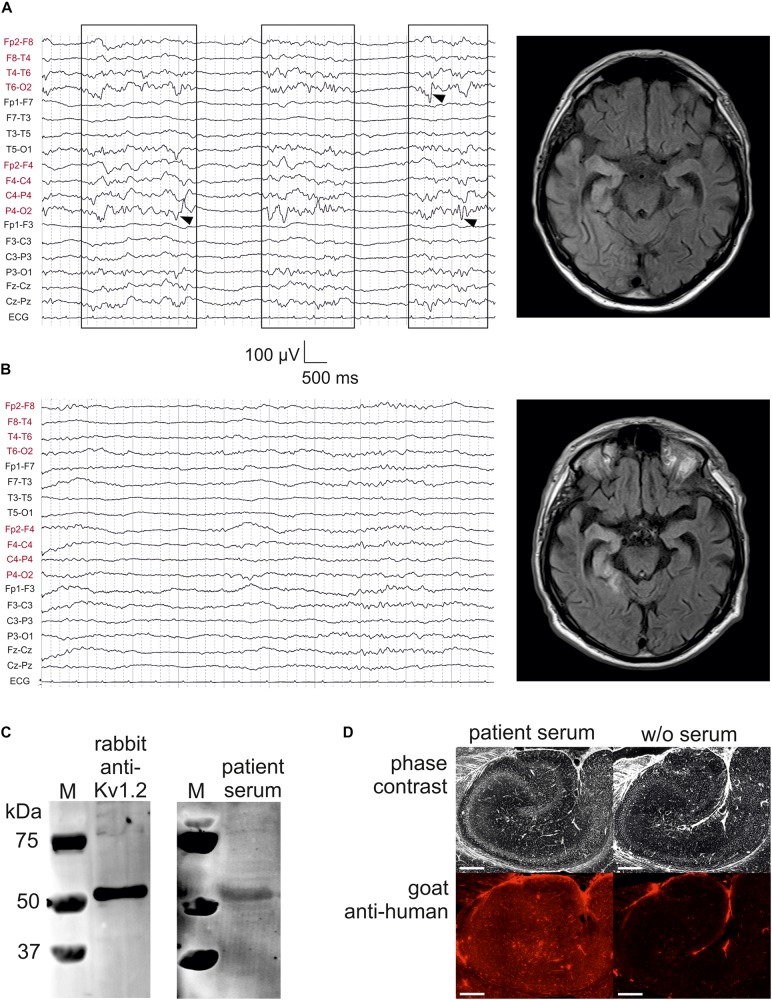
Kv1.2-associated limbic encephalitis. **(A)** EEG and FLAIR MRI on the day of admission, but after starting sedation with propofol. There were bursts of right-sided delta activity and sharp transients (boxes with arrowheads) interrupted by nearly isoelectric lines, consistent with the hyperintensities in right temporoparietal structures involving the mesial temporal lobe. **(B)** EEG and FLAIR MRI after 7 days of treatment at the intensive care unit. Note the low-voltage burst-suppression-like pattern indicating therapeutic coma. On the MRI, hyperintensive lesions have concentrated to temporomesial structures. **(C)** Western blot analysis of rat CA1 protein lysate stained with rabbit anti-Kv1.2 (left panel) and patient serum (right panel). M, marker. **(D)** Specific binding of patient serum serum (left panel) was also confirmed as compared to negative control (w/o serum, right panel). Scale bar = 500 μm.

A 74-year-old male patient was admitted to our emergency with the history of at least two epileptic seizures witnessed by the prehospital emergency medical services. One of these seizures was reported to have presented with a focal onset (orofacial motor symptoms and gaze preference to the left side). Upon initial neurological assessment, he was comatose (Glasgow Coma Scale 3) most likely due to status epilepticus and presented with tetraparesis, but accentuated at the left side. Therefore, he immediately received endotracheal intubation and mechanical ventilation. Computed tomography was inconclusive, the further clinical and paraclinical work-up revealed inflammation (leukocytes 9500/μl; C-reactive protein 90 μmol/l) which was suspected to be associated with aspiration pneumonia, but electrolytes as well as renal and hepatic function did not explain the patient’s clinical situation. The subsequently performed MRI found hyperintense cortices in the right hemisphere, in particular the temporal lobe which was attributed to status epilepticus or encephalitis (Figure 1). The EEG showed epileptic discharges on right hemispheric leads (Figure 1). Hence, we started to treat the patient with levetiracetam and propofol for sedation. Analysis of the cerebrospinal fluid showed increased protein and lactate levels, but could not evidence an acute infectious inflammation (leukocytes 1/μl, lactate 4 mmol/l, protein 520 mg/l, albumin 370 mg/l, negative for intrathecal immunoglobulin synthesis, identical oligoclonal bands in CSF and serum, i.e., type IV reaction). Neurotropic viruses (HSV, VZV, CMV, and EBV) as well as lyme neuroborreliosis were also ruled out. Given the suspected LE, we tested a battery of autoantibodies and found a positive titer for antibodies against VGKCs (115 pmol/l). Unexpectedly, anti-LG1 was negative, but a novel antibody, IgG against the pore-forming subunit KCNA2 (also called Kv1.2), could be detected (titer 1:10, Euroimmun, Lübeck, Germany). The patient received five episodes of plasmapheresis, and subsequently recovered from comatose state and spontaneously opened his eyes. We received blood serum samples together with the permission to keep these samples in −20°C for further scientific studies. The patient was supplied with a percutaneous endoscopic gastrostoma as well as a tracheostoma and was successfully transferred to rehabilitation 4 weeks after admission.

### Case of Anti-CASPR2 Encephalopathy

A 1-year and 8 months old boy was admitted for change of behavior with unprovoked crying, suspected pain, and severe sleeplessness. He developed treatment refractive hypertonia and general weakness. Brain MRI, whole body MRI and laboratory work-up was unremarkable. Cerebrospinal fluid analysis showed mild pleocytosis (12 leucocytes, normal protein, no oligoclonal bands). Neurotropic viruses and neuroborreliosis were ruled out. Testing for antineural antibodies revealed high titer anti-CASPR2 (1:1280) in serum only (VGKC 119 pmol/l). Electrophysiological studies with nerve conductions studies and EEG were normal. Pediatric Morvan syndrome was diagnosed and he received treatment with intravenous immunoglobulins and repeated methylprednisolone pulses which resulted in resolution of acute symptoms. At 3 years of age, he was diagnosed with autism spectrum disorder. The patient’s clinical and serological findings were reported within a series of children with CASPR2 autoimmunity (Syrbe et al., 2020).

### Stereotactic Intrahippocampal Serum Injection *in vivo*

Stereotactic injection of the serum from the case patients or a non-epileptic control subject into both hippocampi *in vivo* was performed as previously described (Blome et al., 2018; Kersten et al., 2019). Briefly, 75 male Wistar rats (8–10 weeks old, Charles River, Sulzfeld, Germany) were anesthetized with S-ketamine (100 mg/kg i.p.) and xylazine (15 mg/kg i.p.) and mounted on a stereotactic frame (Narishige, Tokyo, Japan). Using a Hamilton syringe (75N; Hamilton AG, Bonaduz, Switzerland) patient or control serum was slowly (10 steps of 0.5 μl every 2 min, total of 5 μl for each side) injected into the hippocampus with the following coordinates: 5.2 mm posterior, ±4.3 mm lateral, 4.8 mm deep (relative to bregma). These coordinates were obtained in previous studies (Blome et al., 2018; Kersten et al., 2019). After completing the injection, the syringe remained *in situ* for another 2 min to enable proper serum diffusion into the hippocampus. After surgery, rats were given metamizole (100–150 mg/kg) for postoperative pain control and allowed to recover in an atmosphere with enhanced oxygen fraction (4–5 l/min in an 8 l glass vessel). There was one rat (anti-Kv1.2 group) showing severe respiratory insufficiency and was thus killed during anesthesia, but no further severe morbidity or mortality was observed (overall lethality 1/98). Due to the randomization process, rats treated with anti-Kv1.2, anti-CASPR2 or control serum did not differ significantly in weight during surgery (anti-Kv1.2: 256 ± 10 g, *n* = 36; control: 265 ± 10 g, *n* = 39; anti-CASPR2: 298 ± 12 g, *n* = 23) or in latency between surgery and slice preparation (anti-Kv1.2: 2.6 ± 0.3 days; control: 3.1 ± 0.4 days; anti-CASPR2: 3.6 ± 0.5 days). Moreover, experimenters were blinded to the injected serum (whether anti-Kv1.2, anti-CASPR2 or control serum). In addition, we also used 11 naive, non-operated rats for input–output relations and long-term zero Mg^2+^ experiments as a second control. All procedures were performed according to national and international guidelines on the ethical use of animals (European Council Directive 86/609/EEC, approval of local authority LALLF M-V/TSD/7221.3-1.1-017/11 and M-V/TSD/7221.3-1.1-007/16), and all efforts were made to minimize animal suffering and to reduce the number of animals used.

### Immunodetection of Anti-Kv1.2 in Patient Serum

The immunoreactivity of patient serum was tested by immunofluorescence and Western blot analysis. To this end, hippocampal brain slices of adult male Wistar rats were obtained and either used for immunofluorescence or Western blot. In order to increase the protein content of the IgG fraction, the patient serum was concentrated by a factor of 4 with centrifugal concentrators (Vivaspin) using a 100 kDa molecular weight cutoff filter to omit the albumin fraction. For Western blot analysis, the hippocampal tissue was homogenized in RIPA buffer to disrupt cells. The crude extract was centrifuged to yield a clear protein solution. Performing SDS gel electrophoresis, a total of 15 μg proteins were separated and blotted onto PVDF membranes (Immobilon-FL, Millipore). Overnight incubation with a commercial rabbit polyclonal anti-Kv1.2 antibody (1:1000, Alomone, #APC-010) or patient serum was followed by secondary antibody reaction (anti-human or anti-rabbit IRDye 800CW, Odyssey). Specific proteins band were visualized using the Odyssey infrared imaging scanner (Li-cor). For immunofluorescence staining, the slices were fixed in 3.7% formaldehyde solution, then cryo-preserved with 30% buffered sucrose solution at 4°C overnight and finally frozen. Thin layers of 50 μm thickness were sliced using a cryo-vibratome and mounted on microscope object carriers. Immunoreaction was carried out with or without patient serum (negative control). Fluorescence signals emerged from Cy5-coupled secondary antibodies (goat anti-human, Invitrogen) and were visualized using a Leica DMI 6000B fluorescence microscopy under identical conditions. Each image was taken in a tile scan mode to receive an overview picture from the hippocampus.

### Electrophysiological Recordings and LTP Induction

Hippocampal slices were prepared 1 to 6 days after stereotactic surgery (Blome et al., 2018; Kersten et al., 2019). Briefly, rats were decapitated in deep anesthesia with diethyl ether, the brains were rapidly removed and submerged into oxygenated ice-cold dissection solution containing (in mM) 125 NaCl, 26 NaHCO_3_, 3 KCl, 1.25 NaH_2_PO_4_, 0.2 CaCl_2_, 5 MgCl_2_ and 13 D-glucose (95% O_2_, 5% CO_2_; pH 7.4; 306–314 mosmol/kg). Horizontal hippocampal brain slices (400 μm) were cut using a vibratome (Campden Instruments, Loughborough, United Kingdom), and slices were then transferred into a holding chamber containing artificial cerebrospinal fluid (ACSF) containing (in mM) 125 NaCl, 26 NaHCO_3_, 3 KCl, 1.25 NaH_2_PO_4_, 2.5 CaCl_2_, 1.3 MgCl_2_ and 13 D-glucose (306–314 mosmol/kg, bubbled with 95% O_2_ and 5% CO_2_ to maintain the pH at 7.4).

Synaptic transmission and plasticity were assessed by recording field excitatory postsynaptic potentials (fEPSPs) from CA1 or from the dentate gyrus. The slices were continuously bathed in oxygenated ACSF (flow rate of 2 ml/min, temperature 32 ± 1°C, npi electronic GmbH, Tamm, Germany). For stimulation of the afferent fibers (Schaffer collaterals, or medial perforant path, respectively), bipolar stimulating electrodes fabricated from teflon-insulated platinum wire electrodes (PT-2T, Science Products, Hofheim, Germany) were placed into CA1 stratum radiatum or dentate gyrus middle molecular layer, respectively. Stimuli were delivered through a stimulus isolator (A365, World Precision Instruments, Sarasota, FL, United States) triggered by a Master-8 stimulator (A.M.P.I., Jerusalem, Israel), and the stimulus intensity was routinely increased from 25 to 300 μA. For the prospective analysis of fEPSP slopes (Figure 3C), we adjusted the stimulus intensity in each slice to the half-maximum amplitude, which was typically around 85 μA (between 75 and 125 μA). The same procedure was done to establish the baseline stimulus intensity for LTP experiments. NMDAR-dependent LTP was induced by a paradigm consisting of 10 trains of 20 stimuli at 100 Hz (stimulus duration 100 μs, intertrain interval 800 ms, at double baseline stimulation intensity, Blome et al., 2018; Kersten et al., 2019). NMDAR-independent LTP was induced by a similar paradigm (stimulus duration 100 μs, intertrain interval 800 ms, at double baseline stimulation intensity), but in the presence of D-AP5 (50 μM). Recording electrodes filled with ACSF were placed into CA1 stratum radiatum or dentate gyrus middle molecular layer, respectively. Analog recording signals were amplified, filtered at 1 kHz by an EXT-10-2F (npi electronic GmbH, Tamm, Germany), and digitized with a Micro1401 analog-to-digital converter (Cambridge Electronic Design, Cambridge, United Kingdom) using Signal 2.16 software (Cambridge Electronic Design, Cambridge, United Kingdom). Chemicals used for physiological solutions were purchased from Sigma-Aldrich (Taufkirchen, Germany).

Intracellular recordings were performed in CA1 pyramidal cells impaled with borosilicate glass microelectrodes (80–120 MΩ, pulled with Sutter P-97 and filled with 3 M potassium acetate and 0.013 M KCl) using an SEC-10LX amplifier (npi electronic). In these recordings, RMP was determined, and action potentials were elicited by short (duration 7 ms, from +0.5 to +2.4 nA in 0.1-nA-steps, interstimulus-interval 1.1 s) or prolonged current injections (duration 600 ms, from −1.0 to +1.0 nA in 0.1-nA-steps, interstimulus interval 10 s). Action potential (AP) threshold was assessed as the voltage at the point of the steepest slope of the AP upstroke (i.e., the beginning of the upstroke). We also determined AP amplitude, AP overshoot, and AP duration at half-maximum amplitude (width at half), and the afterhyperpolarizing potential following prolonged depolarization (600 ms, +1.0 nA). Digitization at 10 kHz and offline data processing were performed with the CED package (Micro1401 analog-to-digital converter, Signal 2.16 software, all from Cambridge Electronic Design, Cambridge, United Kingdom).

### Acutely Induced Epileptiform Discharges

In order to test the effect of anti-Kv1.2 on acutely induced epilepsy, we evoked spontaneous epileptic discharges by removal of Mg^2+^ from the bath. In these experiments, the extracellular electrode was placed in the upper blade of the dentate gyrus molecular layer. At the beginning of each experiment, the absence of spontaneous epileptic discharges was confirmed by recording under baseline conditions for at least 20 min. Then Mg^2+^ was omitted from the ACSF, and the recordings were maintained for further 60 min. Spontaneous epileptic events were counted in each slice for the entire recording period of 80 min. Occasionally, epileptic events consisted of more than one spike discharge. In a second approach, these polyspike discharges were analyzed individually. Further events, such as seizure-like episodes or spreading depressions, were also documented.

### Statistical Analysis

Data are expressed as mean values ± the standard error of the mean (SEM) or box-whisker plots (created with SigmaStat 3.5: box = quartiles, whiskers = 10/90% percentiles). For statistical evaluation, data were first tested for normal distribution and equal variance (SigmaStat 3.5). Depending on this normality test, statistical comparisons were performed either using parametric (paired or unpaired *t*-test, ANOVA) or non-parametric tests (Mann–Whitney test) as indicated. The level of significance is indicated by asterisks (^∗^*P* < 0.05, ^∗∗^*P* < 0.01).

## Results

We present a case with status epilepticus due to anti-Kv1.2-associated LE, presenting with lateralized delta activity as well as sharp transients, predominantly over right posterior hemispheric leads (Figure 1A, left panel). Bursts of focal slowing and epileptiform potentials (boxes with arrowheads in Figure 1A) were interrupted by nearly isoelectric lines as a result from propofol medication in the mechanically ventilated patient (burst-suppression pattern). FLAIR MRI at this time point clearly showed high signal intensities on the right temporoparietal lobes with strong involvement of temporomesial structures (Figure 1A, right panel), consistent with the right-sided epileptic activity. One week later, these hyperintense lesions tended to concentrate on these temporomesial structures providing *post hoc* evidence for the LE (Figure 1B, right panel). The EEG at this time point showed typical burst-suppression due to therapeutic coma rather than lateralized epileptic activity (Figure 1B, left panel).

To test the specificity of the patient serum, we performed a Western blot analysis using rat CA1 hippocampal protein lysate immunoblotted with rabbit anti-Kv1.2 which detected a band consistent with the expected molecular weight of rat Kv1.2 (499 amino acids, 57 kDa; Figure 1C, left panel). When this primary antibody was replaced by concentrated albumin-free patient serum counterstained by an anti-human secondary antibody, the same band was obtained (Figure 1C, right panel). Importantly, there were no further bands in this blot suggesting that the predominant protein identified by the patient serum presumably represented Kv1.2. Immunohistochemistry on a rat hippocampal section confirmed that albumin-free patient serum (Figure 1D, left panel) showed specific binding as compared to the negative control without serum (Figure 1D, right panel).

### Pre- and Postsynaptic Excitability and Plasticity

**FIGURE 2:**
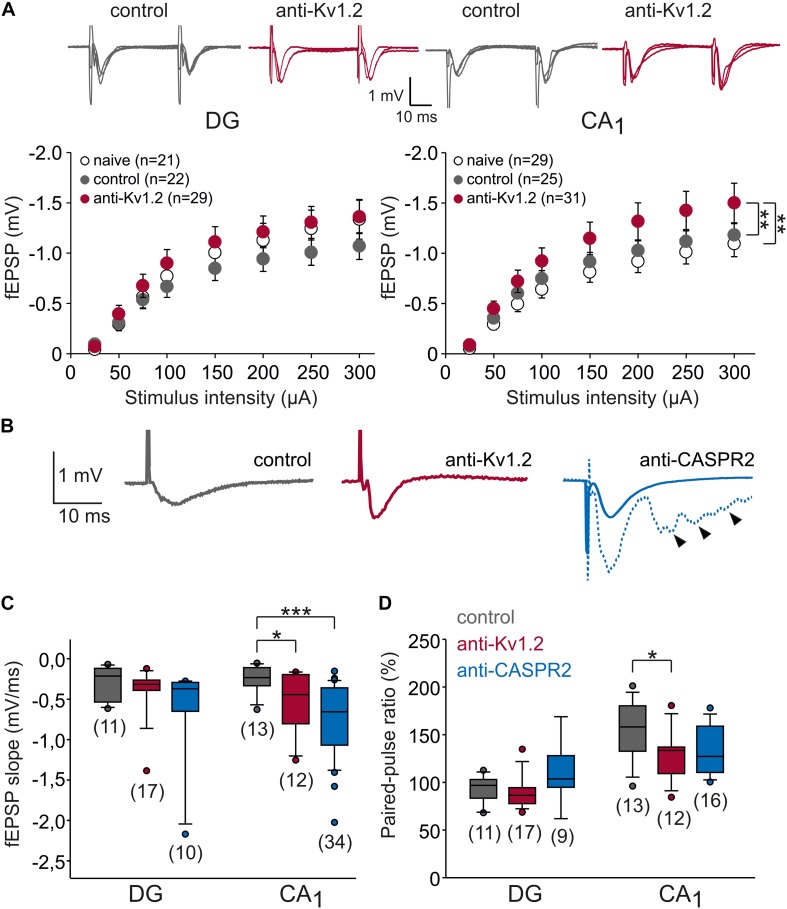
Synaptic transmission is altered in CA1, but not the dentate gyrus. **(A)** Input–output relationships of dentate gyrus (DG) and CA1 area of the hippocampus. Sample traces were obtained by increasing stimulation intensities (100, 200, 300 μA). In CA1, but not in the dentate gyrus, fEPSPs from anti-Kv1.2 tissue (red symbols) were significantly different from those obtained in slices of control-injected (gray symbols) or non-operated rats (open symbols). Statistical comparison was performed using two-way ANOVA and Tukey *post hoc* tests (factors stimulus intensity and animal group). **(B)** Representative recordings of fEPSPs in the CA1 area showing the more negative slope in anti-Kv1.2 tissue (red trace) and anti-CASPR2 tissue (blue trace) as opposed to control-injected tissue (gray trace). Note that in anti-CASPR2 tissue, a proportion of cells showed epileptic afterdischarges at baseline (arrowheads in dashed blue trace). **(C)** Box-whisker plots of fEPSP slopes at the half-maximum size show significant differences in CA1 (*P < 0.05, Student *t*-test; ***P < 0.001, Mann–Whitney *U* test), but not in the dentate gyrus. **(D)** Box-whisker plots of paired-pulse ratio (PPR) show significant differences between anti-Kv1.2 and control in CA1 (*P < 0.05, Mann–Whitney *U* test), but not in the dentate gyrus.

In this study, we aimed to study the effects of patient serum containing anti-Kv1.2 on synaptic function. To this end, we stereotactically injected serum into the hippocampus *in vivo* and obtained horizontal hippocampal brain slices 1–6 days after surgery. We studied the Schaffer collateral-CA1 synapse and the medial perforant path-dentate gyrus synapse, both typical expression sites of Kv1.2. Increasing stimulation intensities (25 to 300 μA) of the afferent fibers led to increasing excitatory postsynaptic field potentials (fEPSPs) in the respective hippocampal area (Figure 2A). Obviously, two-way ANOVA revealed a significant effect of stimulus intensity (*P* < 0.01 in both fields), but there was no significant interaction between stimulus intensity and animal group (*P* < 0.9 in both fields). While input–output relations did not show consistent significant differences for anti-Kv1.2 in the dentate gyrus (Tukey *post hoc* test: *P* < 0.05 between control and anti-Kv1.2, but *P* = 0.429 between naive and anti-Kv1.2), there was a consistent group effect in the CA1 area (Tukey *post hoc* test: *P* = 0.01 between anti-Kv1.2 and control as well as between anti-Kv1.2 and naive tissue, Figure 2A). Since we observed that the slope of the fEPSP recorded in CA1 was more negative in slices from anti-Kv1.2-treated than in control-injected rats (Figure 2B), we performed a further series of experiments and selected the half-maximum fEPSP of each experiment in order to determine the slope of this potential. As shown in Figure 2C, the negative slope of the half-maximum fEPSP in anti-Kv1.2-treated tissue was indeed significantly higher than in controls at Schaffer-collateral-CA1 synapses (anti-Kv1.2: *P* < 0.05, Student *t*-test), but not in the dentate gyrus (*P* = 0.323, Mann–Whitney *U* test). Since we aimed to compare anti-Kv1.2 effects with anti-CASPR2 effects, we used a further patient serum containing CASPR2 autoantibodies. In anti-CASPR2-treated tissue, the slope of the fEPSP was highly variable and, occasionally, we obtained fEPSPs with epileptic afterdischarges (38% of anti-CASPR2 slices, see arrowhead in rightmost traces, Figure 2B). Hence, the negative slope of the half-maximum fEPSP in this tissue was significantly higher than in control slices (*P* < 0.001, Mann–Whitney *U* test, Figure 2C) indicating enhanced synaptic transmission under both pathological conditions, but suggesting differences between anti-Kv1.2 and anti-CASPR2.

Increased fEPSP slopes may suggest a more synchronous transmitter release, for instance by an inhibitory effect of anti-Kv1.2 on presynaptic terminals, because Kv1 channels were reported to dampen presynaptic post-firing hyperexcitability (Dodson et al., 2003). One measure to address this question is to test paired-pulse plasticity. Since transmitter release probability is inversely related to the paired-pulse ratio (PPR; Zucker, 2002), we delivered paired pulses (interstimulus interval 40 ms). As expected, the PPR of fEPSP slopes in the dentate gyrus was not significantly altered (anti-Kv1.2: 89 ± 4%, *n* = 16; control: 92 ± 4%, *n* = 11; *P* = 0.608; Mann–Whitney *U* test; Figure 2D), but confirmed the typical paired-pulse depression in the medial perforant path (Dietrich et al., 1997). In contrast, slices from anti-CASPR2-treated animals showed paired-pulse facilitation rather than depression (110 ± 9%, *n* = 9, Figure 2D) which could suggest that anti-CASPR2 leads to presynaptic inhibition at medial perforant path terminals. In contrast, we obtained significantly reduced PPR of fEPSP slopes in the CA1 area from anti-Kv1.2 tissue as compared to control (anti-Kv1.2: 129 ± 7%, *n* = 12; control: 153 ± 8%, *n* = 13; *P* < 0.05, Mann–Whitney *U* test; Figure 2D), suggesting an increased transmitter release probability at these synapses. The PPR in anti-CASPR2 slices was only slightly reduced (133 ± 6%, *n* = 16, *P* = 0.076 versus control, Mann–Whitney *U* test; Figure 2D) indicating that the increased fEPSP slope in this tissue was partially due to increased presynaptic transmitter release, but may have involved other mechanisms such as network disinhibition.

So far, these data suggest that anti-Kv1.2 and anti-CASPR2 differ in their effects on Schaffer collateral presynaptic function. However, they cannot exclude that postsynaptic cells may also be altered in anti-Kv1.2 or anti-CASPR2 tissue. To control for this, we performed intracellular recordings of CA1 neurons from anti-Kv1.2, anti-CASPR2 and control rats. At first, there were no significant differences in the RMP and cellular membrane properties such as resistance and time constant between anti-Kv1.2 and controls (Table 1), indicating that anti-Kv1.2 did not significantly affect the leak conductance. When we applied short current injections to elicit a single action potential (duration 7 ms, interstimulus interval 1.1 s), there were again no differences in action potential characteristics (Table 1) arguing against major postsynaptic differences in intrinsic cellular excitability between these two groups. In contrast, cells from anti-CASPR2 tissue differed from cells recorded in control or anti-Kv1.2-treated slices (Table 1). It is important to note, however, that all significant differences obtained (especially action potential characteristics and afterhyperpolarizing potential) are not compatible with an impaired K^+^ channel function of CA1 neurons, but may rather indicate separate changes on the network level.

**TABLE 1:** Intrinsic CA1 pyramidal cell properties.

	Control (*n* = 9)	Anti-Kv1.2 (*n* = 11)	Anti-CASPR2 (*n* = 9)
Resting membrane potential	−58.4 ± 1.2mV	−59.1 ± 1.6mV	−62.5 ± 1.3mV
Membrane resistance	45.7 ± 3.7MΩ	43.1 ± 6.3MΩ	39.7 ± 3.8MΩ
Membrane time constant*	7.3 ± 0.7ms	8.3 ± 0.6ms	10.2 ± 0.8ms (a)
Action potential amplitude	65.9 ± 2.0mV	64.1 ± 2.4mV	67.8 ± 1.3mV
Action potential overshoot	6.6 ± 1.1mV	4.5 ± 2.2mV	3.3 ± 1.8mV
Action potential width at half*	1.6 ± 0.1ms	1.6 ± 0.1ms	1.4 ± 0.05ms (a,b)
Action potential threshold*	−45.1 ± 1.3mV	−43.8 ± 2.4mV	−51.0 ± 1.9mV (a,b)
Afterhyperpolarizing potential*	−6.3 ± 0.9mV	−5.9 ± 1.1mV	−13.6 ± 1.5mV (a,b)

*Statistical comparisons were performed using ANOVA with Student–Newman–Keuls post hoc tests. Significant group effects are indicated with asterisks, the lowercase letters in parentheses indicate statistically significant differences between anti-CASPR2 and control (a), or between anti-CASPR2 and anti-Kv1.2 (b).*

**FIGURE 3:**
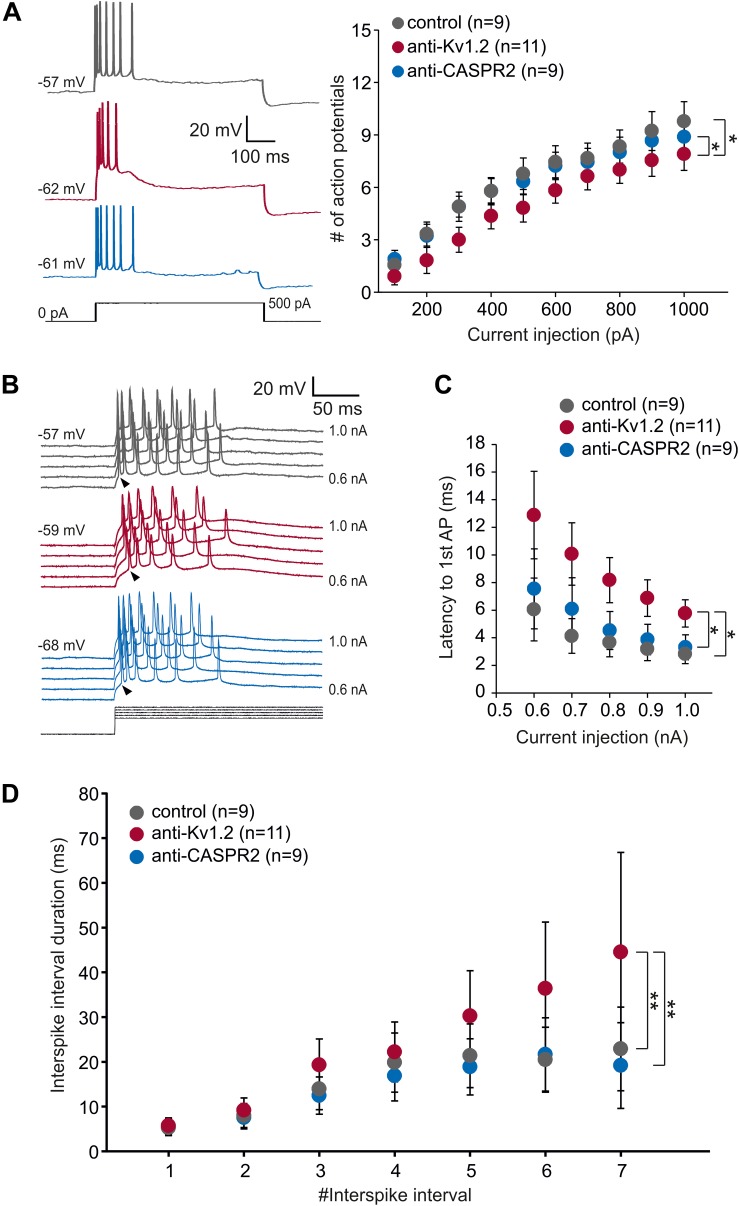
Intrinsic properties of CA1 neurons. **(A)** Representative intracellular voltage recordings of a CA1 neuron from anti-Kv1.2 (red traces), control-injected (gray traces) and anti-CASPR2 (blue traces) animals evoked by prolonged current injection (500 pA, black trace). Two-way ANOVA and Tukey *post hoc* tests (factors current injection and animal group) detected a significant effect of animal group between anti-Kv1.2 and control (*P < 0.05) as well as between anti-Kv1.2 and anti-CASPR2 (*P < 0.05) regarding the number of action potentials elicited by depolarizing current injection (600 ms). Importantly, while there was a significant effect of current injection (*P* < 0.05), there was no significant interaction between current injection and animal group (*P* = 0.991). **(B)** Voltage recordings from CA1 neurons evoked by increasing steps of suprathreshold prolonged current injection (0.5 to 1.0 nA in 0.1-nA-steps, black traces). Arrowheads point to the onset of the first action potential which was delayed in anti-Kv1.2 tissue (red traces) as compared to control (gray traces) and anti-CASPR2 (blue traces). **(C)** The latency to the 1st AP was significantly delayed in anti-Kv1.2 tissue compared to both other groups (*P < 0.05, two-way ANOVA and Tukey *post hoc* test). **(D)** Interspike interval duration increases during the course of depolarization due to spike frequency adaptation. Cells from anti-Kv1.2 tissue showed significantly prolonged interspike interval durations compared to both other groups (**P < 0.01, two-way ANOVA and Tukey *post hoc* test).

In addition, one unique property of Kv1.2, but not of other Kv1 channels, is the use-dependent activation (Baronas et al., 2015). Hence, we prolonged the current injection to 600 ms (500 pA) with an interstimulus interval of 10 s. Under these conditions, we observed that CA1 cells from anti-Kv1.2 showed a significantly reduced number of action potentials compared to control (*P* < 0.05, two-way ANOVA with factors current injection and animal group, followed by Tukey *post hoc* test; Figure 3A). Importantly, there was also a significant difference between anti-Kv1.2 and anti-CASPR2 (*P* < 0.05; Figure 3A), while there was no significant interaction between current injection and animal group (*P* = 0.991, two-way ANOVA). However, spike broadening was not different between all experimental groups as assessed by the action potential duration (width at half: 3rd AP relative to 1st: 168 ± 51%, *n* = 11 in anti-Kv1.2; 178 ± 59%, *n* = 9 in control; 176 ± 59%, *n* = 9 in anti-CASPR2; *P* = 0.369, one-way ANOVA).

Intriguingly, we also noticed that the first action potential occurred later in anti-Kv1.2-treated than in control tissue (see arrowheads in Figure 3B). This was a consistent finding, CA1 neurons from anti-Kv1.2 tissue showed significantly higher latencies to the first action potential compared to both control and anti-CASPR2 tissue (*P* < 0.05, two-way ANOVA with factors current injection and animal group, followed by Tukey *post hoc* test; Figure 3C). In addition, we analyzed the interspike interval duration during current injection (600 ms, +1.0 nA) and found significantly prolonged interspike intervals in cells from anti-Kv1.2-treated rats as compared to both other groups (*P* < 0.01 versus control and anti-CASPR2, two-way ANOVA with factors #interspike interval and animal group, followed by Tukey *post hoc* test; Figure 3D). Since the delayed firing in anti-Kv1.2 could not be attributed to an altered membrane time constant (Table 1), our data suggest that both pre- and postsynaptic sites may be involved in changes observed in anti-Kv1.2 tissue.

**FIGURE 4:**
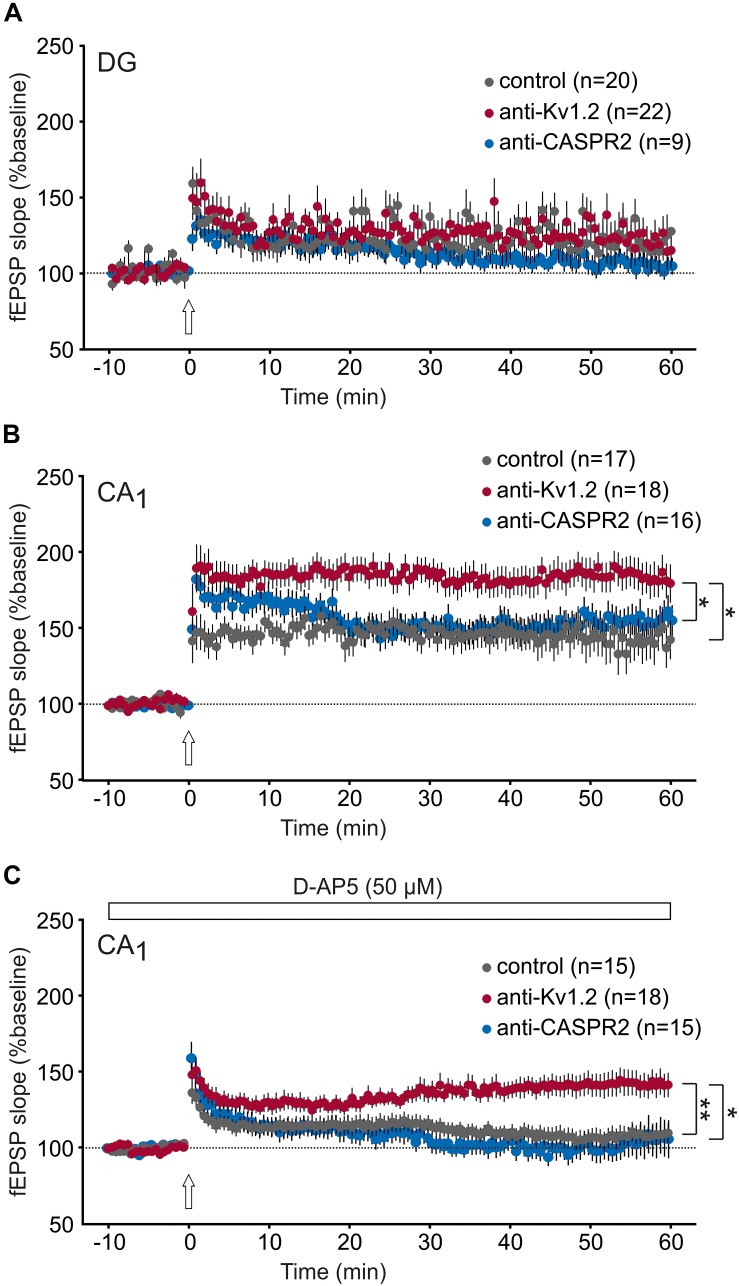
Synaptic plasticity is enhanced in CA1, but not the dentate gyrus. **(A)** Long-term potentiation (LTP) in the dentate gyrus (DG) was not different between anti-Kv1.2, anti-CASPR2 and control slices. The arrow indicates the time-point of LTP inducing high-frequency stimulation. **(B)** LTP in the CA1 area reveals a significant increase in anti-Kv1.2 tissue compared to control (*P < 0.05, Student *t*-test) and anti-CASPR2 (*P < 0.05, Mann–Whitney *U* test). **(C)** Slices from anti-Kv1.2-treated rats also showed enhanced NMDAR-independent LTP in CA1 (**P < 0.01 versus control, Student *t*-test; *P < 0.05 versus anti-CASPR2, Student *t*-test). The entire experiment was carried out in the presence of the NMDAR blocker D-AP5 (50 μM).

Next, we asked whether the level of achievable long-term potentiation (LTP) might be altered in anti-Kv1.2 tissue. Thus, we were interested in the propensity to activate postsynaptic NMDA receptors upon high-frequency extracellular afferent stimulation. Consistent with our previous data so far, LTP at the medial perforant path-dentate gyrus synapse was almost identical between all three groups (anti-Kv1.2: 120 ± 11%, *n* = 22; control: 120 ± 13%, *n* = 20; anti-CASPR2: 104 ± 5%, *n* = 9; Figure 4A), but in marked contrast, CA1-LTP in anti-Kv1.2-treated tissue (182 ± 11%, *n* = 18) was significantly higher as compared to both control (143 ± 14%, *n* = 17, *P* < 0.05, Student *t*-test) and anti-CASPR2 tissue (155 ± 10%, *n* = 16, *P* < 0.05, Mann–Whitney *U* test; Figure 4B). In addition, we also tested NMDAR-independent LTP at Schaffer collateral-CA1 synapses which was again significantly enhanced in slices from anti-Kv1.2-treated rats (143 ± 10%, *n* = 18; control: 109 ± 6%, *n* = 15, *P* < 0.01, Student *t*-test; anti-CASPR2: 106 ± 13%, *n* = 15, *P* < 0.05, Student *t*-test; Figure 4C). Taken together, these findings suggest that stereotactic injection with patient serum containing anti-Kv1.2 facilitates presynaptic transmitter release and postsynaptic depolarization at the Schaffer collateral-CA1 synapse, but not in the dentate gyrus, and as a consequence of this, both synaptic transmission and LTP in CA1 are facilitated. In contrast, patient serum containing anti-CASPR2 enhances postsynaptic responses, but rather independently of K^+^ channel modulation, and does not interfere with LTP at Schaffer collateral-CA1 synapses.

### Vulnerability to Epileptic Conditions

**FIGURE 5:**
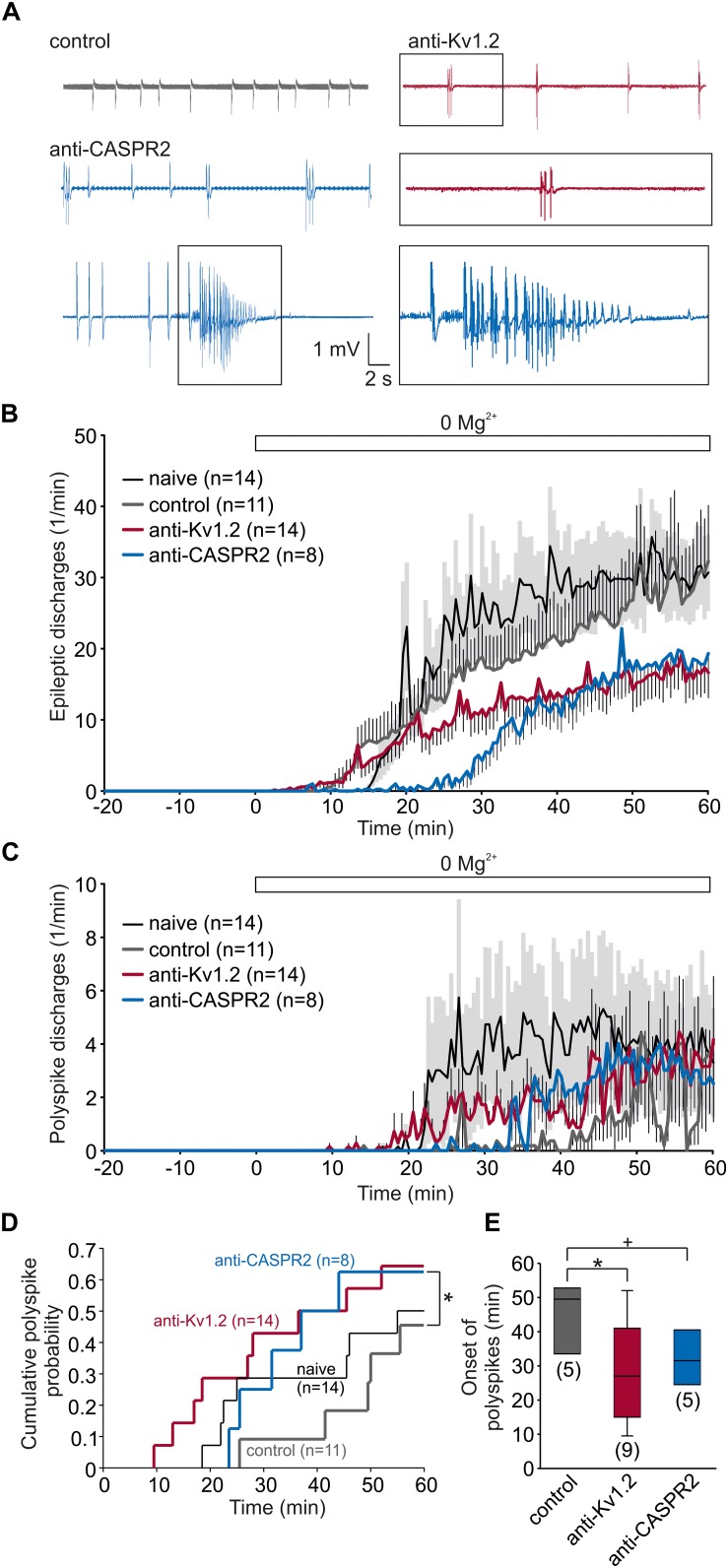
Susceptibility to pro-epileptic conditions is altered in anti-Kv1.2 tissue. **(A)** Representative recordings from slices under pro-epileptic conditions (removal of Mg^2+^). Note the polyspike discharge in the anti-Kv1.2 trace (red), shown enlarged in the box below. In anti-CASPR2 tissue, there were again polyspike discharges, but also seizure-like events (lowermost row, enlarged in the box). **(B)** Time course of epileptic discharges in slices from non-operated rats (black), anti-Kv1.2-treated rats (red), anti-CASPR2-treated rats (blue) and control-injected rats (gray). Mg^2+^ was omitted from the bath at time-point 0 (indicated by the open bar). SEM for anti-Kv1.2, anti-CASPR2 and control tissue is depicted as antennas; SEM for non-operated rats is depicted as a light gray area. Note that epileptic discharges in slices from anti-Kv1.2 tissue were less frequent than in both control groups. **(C)** Time course of polyspike discharges in all experimental groups. Note the difference in polyspike discharge onset between slices from anti-Kv1.2 and anti-CASPR2 or control-injected rats. **(D)** Cumulative probability plots of polyspike discharge incidence rates reveal a difference between anti-Kv1.2 and control (*P* = 0.109, Kaplan–Meier log-rank test) as well as between anti-CASPR2 and control (*P = 0.05, Kaplan–Meier log-rank test). **(E)** Onset of polyspike dischargeswhen they were present. The difference in polyspike discharge onset was still significant between anti-Kv1.2 and control (^+^*P* = 0.095, *P < 0.05, Mann–Whitney *U* test).

Facilitation of synaptic transmission and plasticity may imply that the tissue might be more vulnerable to hyperexcitable states. Since the anti-Kv1.2 encephalitis suffered from epilepsy, we tested the susceptibility of anti-Kv1.2-treated and anti-CASPR2-treated tissue by removal of Mg^2+^ from the bath. Under these conditions, spontaneous epileptiform potentials were elicited in all experimental groups (Figure 5A). In fact, there was a tendency toward more epileptic events in control tissue, albeit not significant (Figure 5B). While the vast majority of epileptic events consisted of a single interictal spike, we occasionally observed two or more spikes (referred to as polyspike discharges) and, intriguingly, the incidence of these polyspike discharges was markedly higher in slices from anti-Kv1.2-treated and anti-CASPR2-treated rats (Figure 5C). In order to quantify the development of polyspike discharges, we determined the cumulative probability of polyspike discharge incidence rates showing a trend toward a shorter latency and a higher proportion in anti-Kv1.2 tissue (*P* = 0.109, Kaplan–Meier log-rank test) and anti-CASPR2 tissue (*P* = 0.05, Kaplan–Meier log-rank test, Figure 5D). When only slices with polyspike discharges were analyzed, the onset of these discharges was significantly advanced in anti-Kv1.2 as compared to control (*P* < 0.05, Mann–Whitney *U* test; Figure 5E). These data indicate that susceptibility to epileptic conditions was altered in anti-Kv1.2 tissue. The onset of polyspike discharges in anti-CASPR2 tissue tended to be also advanced (*P* = 0.095, Mann–Whitney *U* test; Figure 5E), but more intriguingly, this tissue was markedly prone to seizure-like events (see blue sample traces in Figure 5A) which were never observed in the other groups (anti-CASPR2: 4/8 slices, *P* < 0.05 versus anti-Kv1.2 and control, Fisher’s exact test).

## Discussion

We present a case of Kv1.2-associated LE with status epilepticus. We took advantage of testing this patient’s serum by stereotactic injection into the rat hippocampus. The human Kv1.2 protein primary structure is 99.4% identical to the rat isoform (NCBI, blast analysis). Therefore, we assumed that the human antibody could bind to the rat Kv1.2 in the hippocampus, and using Western blot we showed that both a commercial rabbit anti-Kv1.2 and the patient’s serum detected the same band which was consistent with the calculated molecular weight of 57 kDa for this protein. We also had the opportunity to compare this patient’s serum with a serum from a case of anti-CASPR2 encephalopathy. This approach was also used with patient cerebrospinal fluid containing NMDAR or GAD65 antibodies in previous studies (Hackert et al., 2016; Würdemann et al., 2016; Blome et al., 2018; Kersten et al., 2019). Thus, we propose the intrahippocampal bolus injection of serum is also a valid model for studying patient-derived autoantibodies.

Originally, Kv1 shaker-related K^+^ channels have been characterized pharmacologically by the use of dendrotoxins, relatively selective Kv1 inhibitors derived from the venom of *Dendroaspis* snakes (Dolly et al., 1984; Rehm and Lazdunski, 1988; Parcej and Dolly, 1989). Currently, seven pore-forming α-subunits Kv1.1-1.7 have been cloned (Gutman and Chandy, 1993) and studied in various heterologous expression systems revealing substantial differences in activation and inactivation kinetics (Baranauskas, 2007). In addition, auxiliary β-subunits such as Kvβ1-3 with splice variants were discovered which may modify gating properties or serve as chaperones (Rettig et al., 1994; Scott et al., 1994; England et al., 1995; Heinemann et al., 1995; Majumder et al., 1995). The complexity of gating properties, in particular of Kv1.2, is further increased by two different activation kinetics referred to as “slow” and “fast” gating channels (Rezazadeh et al., 2007) and, finally, by glycosylation of the extracellular S1-S2 linker resulting in a depolarizing shift in the half-maximal activation (Watanabe et al., 2007). Typical features of Kv1.2 are the low threshold (below action potential threshold) and the use-dependent activation (Baronas et al., 2015). Thus, Kv1.2 is activated before the action potential is elicited, and repetitive firing may increase Kv1.2 contribution to AP repolarization. It is well established that Kv1.2 is predominantly expressed at presynaptic terminals (Wang et al., 1993, 1994; Sheng et al., 1994; Monaghan et al., 2001; Gu et al., 2003; Wenzel et al., 2007; Lorincz and Nusser, 2008), but functional characterization of presynaptic ion channels is feasible only at highly specialized synapses such as the Calyx of Held (Rezazadeh et al., 2007). While it is actually impossible to predict the effects of antibodies against Kv1.2 at a certain synapse, the available data indicate that Kv1.2 powerfully counteracts depolarizing afterpotentials and thereby constrains transmitter release (Lambe and Aghajanian, 2001; Dodson et al., 2003).

What can we learn from the effects of the patient’s antibody against Kv1.2? We found that Schaffer collateral-CA1, but not medial perforant path-dentate gyrus synapses showed an increased slope of the fEPSP suggesting higher synchronization of transmitter release. In the view of the role of Kv1.2 at presynaptic terminals, this implies the view that Kv1.2 antibodies may exert inhibitory effects on presynaptic Kv1.2 function which was confirmed by the significant reduction of the PPR. A similar finding was obtained from rats after intracerebroventricular injection of Kv1.4-antisense oligodeoxyribonucleotides which led to a reduced PPR and, in addition, impaired LTP in CA1 (Meiri et al., 1998). The latter, however, is difficult to explain, since reduced paired-pulse plasticity should imply an increase of transmitter release probability due to the reduction of presynaptic Kv1.4 channel function. Possibly, LTP in that study was reduced by a concomitant impairment of AMPA receptors as recently reported for anti-LGI1 (Petit-Pedrol et al., 2018). Nonetheless, increased release probability should facilitate postsynaptic depolarization and thereby LTP. In the present study, NMDAR-dependent and NMDAR-independent LTP both relying on postsynaptic depolarization were indeed enhanced in anti-Kv1.2-treated tissue in the present study. Interestingly, LTP in the dentate gyrus was preserved in both the present anti-Kv1.2 model and the Kv1.4-antisense rat (Meiri et al., 1998). Hence, anti-Kv1.2 appears to negatively interfere with presynaptic Kv1.2 function. However, the precise epitope of the antibody and consequently its molecular effects remain open, since antibody binding might affect activation, inactivation or even recovery from inactivation. The latter, for instance, is extremely slow in D-type K^+^ currents (Storm, 1988).

Similar to anti-Kv1.2, experiments with anti-CASPR2 showed an increased slope of the fEPSP in CA1, but not in the dentate gyrus. However, in contrast to Kv1.2, anti-CASPR2 did not significantly reduce the PPR and, moreover, LTP values were indistinguishable from control values. These data indicate that antibodies against CASPR2 which is crucial for axonal targeting of Kv1.2 (Pinatel et al., 2017), does not seem to interfere with presynaptic Kv1.2 function. In addition, these experiments also help argue against putatively unspecific effects of the anti-Kv1.2 patient’s serum, since all data on postsynaptic CA1 action potential firing properties did not differ between control and anti-CASPR2 tissues. It is, however, obvious that we cannot fully exclude that the patient’s serum might contain further agents such as propofol or levetiracetam, but it is unlikely that these compounds would depress K^+^ channel function. A further line of evidence against unspecific effects of anti-Kv1.2 serum derives from the differential effects of this antibody on CA1 and the dentate gyrus. In fact, it was an unexpected and intriguing finding that the changes were restricted to the Schaffer collateral-CA1 synapse. This might be explained by the high diversity of Kv1.2 channel-complexes with auxiliary β-subunits. In this sense, it is important to note that the extracellular S1-S2 linker glycosylation is a relevant regulator of Kv1.2 activation (Watanabe et al., 2007), and *N*-glycosylation was required for the epitope retrieval of patient-derived antibodies in NMDAR encephalitis (Gleichman et al., 2012). Alternatively, it is also possible that the patient’s antibody binds to the VGKC-complex as a whole rather than exclusively the pore-forming α-subunit as previously shown for other VGKC-complex antibody species (Irani et al., 2010; Lai et al., 2010).

It has been shown that Kv1.2 is expressed on CA1 pyramidal cells, but not on dentate granule cells (Sheng et al., 1994; Guan et al., 2006). Therefore, Kv1.2 might contribute to the RMP as well as the action potential (AP) threshold and repolarization. Intracellular recordings from CA1 neurons, however, failed to detect differences in the RMP or AP characteristics such as threshold, amplitude or duration suggesting that Kv1.2 may only play a minor role under resting conditions. In addition, this may also reflect the predominantly dendritic expression of Kv1.2 (Sheng et al., 1994; Guan et al., 2006). In contrast, during prolonged depolarization CA1 neurons from anti-Kv1.2-treated rats showed a delayed onset of the first AP as well as reduced numbers of action potentials. Importantly, the latter finding refers to the original description of D-type K^+^ currents: K^+^ currents with activation thresholds below the AP threshold which therefore delayed the firing were called D-type (i.e., delay-type) (Storm, 1988). In line with this finding, the interspike interval during prolonged depolarization was significantly increased in anti-Kv1.2 tissue. It is important to note that all these findings were specific for anti-Kv1.2, since cells from anti-CASPR2-treated animals showed the same results as controls and therefore significantly differed from cells recorded in anti-Kv1.2 tissue. At first sight, the anti-Kv1.2 effects may suggest facilitated hyperpolarization rather than inhibited K^+^ outward currents. This is difficult to appreciate because differential effects would have to be proposed for the same antibody. However, Kv1.2 channels can be divided into slow and fast gating channels (Rezazadeh et al., 2007), and it is uncertain to what extent gating properties observed in expression systems can be transferred to presynaptic terminals. But for the dendritic Kv1.2 channels together with the use-dependent activation of Kv1.2 (Baronas et al., 2015), it is possible that the patient’s antibody shifts the slow gating channel into the fast gating one. Interestingly, both loss-of-function and gain-of-function mutations of the Kv1.2-encoding gene, KCNA2, were associated with epileptic encephalopathy (Syrbe et al., 2015). This suggests that both hyper- and hypofunction of Kv1.2 can lead to hyperexcitability.

Lastly, we also tested the tissue susceptibility to hyperexcitable states. We found that epileptic events were not significantly different between anti-Kv1.2 and both control groups. However, polyspike discharges occurred significantly earlier and with higher incidence rates in tissue from anti-Kv1.2-treated animals. Intriguingly, anti-CASPR2 tissue also showed higher rates of polyspike discharges, and, even more important, presented with seizure-like events. Together, both patient sera converted the tissue to become more vulnerable to pro-epileptic conditions. Nonetheless, the network changes in anti-Kv1.2 tissue with respect to hyperexcitability were obviously less pronounced when compared to the pre- and postsynaptic changes at Schaffer collateral-CA1 synapses as described before. This could be due to anti-Kv1.2 effects in opposite directions as observed with prolonged depolarizations in intracellular recordings, in particular with respect to hyperexcitability following both hyper- and hypofunction of KCNA2 mutations (Syrbe et al., 2015). In addition, this may also partially be attributed to the fact that Kv1.2 plays a minor role at perforant path-dentate gyrus synapses (present study) as well as at CA3 synapses (Sheng et al., 1994; Prüss et al., 2010).

What can we learn for patient care from these studies? Although it may be uncertain whether the patient’s Kv1.2 antibody binds Kv1.2 directly or not, Kv1.2 appears crucial for governing excitability in the CNS. As known from knockout studies, Kv1.2 deletion is lethal within early postnatal stages, and contrasts with mild consequences of knocking out most other Kv1 channels (London et al., 1998; Smart et al., 1998; Brew et al., 2007), although Kv1.1 is much more widely expressed in the hippocampus than Kv1.2 (Wang et al., 1993, 1994; Veh et al., 1995; Grigg et al., 2000). Nonetheless, Kv1.1 and Kv1.2 are predominant target structures of autoimmune pathology in LE and Morvan syndrome (Kleopa et al., 2006). Hence, our studies can help distinguish clinically relevant autoantibodies from those representing rather as an epiphenomenon such as anti-GAD65 (Stemmler et al., 2015; Widman et al., 2015; Hackert et al., 2016). Moreover, the present study showed that anti-Kv1.2 and anti-CASPR2 differed in their cellular effects, but both led to hyperexcitability. In this sense, both patients’ antibodies should be regarded as pathophysiologically relevant which is in line with previous studies on VGKC complex-antibodies (Lalic et al., 2011; Petit-Pedrol et al., 2018) and thereby give *in vitro* evidence for the clinical decision to use plasmapheresis in the present case.

## Data Availability Statement

The datasets generated for this study are available on request to the corresponding author.

## Ethics Statement

The studies involving human participants were reviewed and approved by the Ethikkommission der Universität Rostock. The patients/participants provided their written informed consent to participate in this study. The animal study was reviewed and approved by the Landesamt für Lebensmittel, Landwirtschaft und Fischerei Mecklenburg-Vorpommern 7221.3-1.1-017/11 and 7221.3-1.1-007/16. Written informed consent was obtained from the individual(s) for the publication of any potentially identifiable images or data included in this article.

## Author Contributions

HB, SK, AG, MW, SS, and AS collected the patient data. TK, ES, GH-G, JB, XG, SM, KP, and TS performed the experiments. TK, AG, MW, SS, AS, and RK contributed conception and design of the study. TK, ES, and GH-G organized the database. TK, XG, D-CH, and KP performed the statistical analysis. TK wrote the first draft of the manuscript. AG, MW, SS, AS, and RK wrote sections of the manuscript. SM, HB, SK, and KP contributed to manuscript preparation. All authors contributed to manuscript revision, read and approved the final version of this manuscript for submission.

## Conflict of Interest

The authors declare that the research was conducted in the absence of any commercial or financial relationships that could be construed as a potential conflict of interest.
